# A 4 and a half years old boy with mesenchymal hamartomas in the left lateral lobe of the liver: A case report and literature review

**DOI:** 10.1097/MD.0000000000007281

**Published:** 2017-08-04

**Authors:** Wei Liao, Binhao Zhang, Wei Zhang, Lin Chen, Wanguang Zhang, Bixiang Zhang, Xiaoping Chen

**Affiliations:** Hepatic Surgery Center, Department of Surgery, Tongji Hospital, Tongji Medical College, Huazhong University of Science and Technology, Wuhan, China.

**Keywords:** 4 years old boy, mesenchymal hamartomas of the liver, surgery, twins

## Abstract

**Rationale:**

Mesenchymal hamartomas of the liver is one type of rare liver tumor.

**Patient concerns:**

Mesenchymal hamartomas of the liver (MHL) is rarely reported in the left lobe of the liver in children who are more than 2 years old. It is difficult to distinguish it from liver lesions such as hepatoblastoma in children, and hepatocellular carcinoma and focal nodular hyperplasia in adults. In addition, it is hard to correctly diagnose it without pathological examination.

**Diagnoses:**

Mesenchymal hamartomas of the liver.

**Interventions:**

This patient underwent an operation assisted by the Da Vinci surgical system and the tumor was completely resected.

**Outcomes:**

No tumor recurrence or metastasis was observed 14 months after operation.

**Lessons:**

MHL is a benign tumor that is difficult to diagnose due to the lack of specific clinical symptoms and signs. The management of MHL remains controversial. To achieve a good long-term outcome, complete resection of MHL is recommended.

## 1 Introduction

Mesenchymal hamartomas of the liver (MHL) was first reported in 1903 by Maresch.^[1]^ Up to 2003, the number of all MHL cases reported in English was less than 200.^[2]^ It is the second most common benign tumor in children within 2 years old.^[3,4]^ However, the tumor is rare in children more than 2 years old and adults. Only 46 adult cases have been reported up to now.^[5]^ About 75% of MHL was found in the right lobe of the liver.^[4]^ The clinical manifestations and radiological examinations of MHL are typically nonspecific. It is often misdiagnosed as other diseases, such as hepatoblastoma (HB) of children, hepatocellular carcinoma (HCC) of adults, or focal nodular hyperplasia (FNH).^[6]^

Here, we report a male child (4 and a half years old) with MHL in the left lateral lobe of the liver. The tumor size was 14 cm × 13 cm × 7 cm and was removed by an operation through the Da Vinci surgical system (DVSS). Interestingly, the patient had an identical twin who was admitted to our hospital to take a magnetic resonance imaging (MRI) test of his liver. The result showed no positive findings. We reviewed and analyzed the literature to provide useful information for doctors to distinguish the disease.

## 2 Methods and results

**Figure 1:**
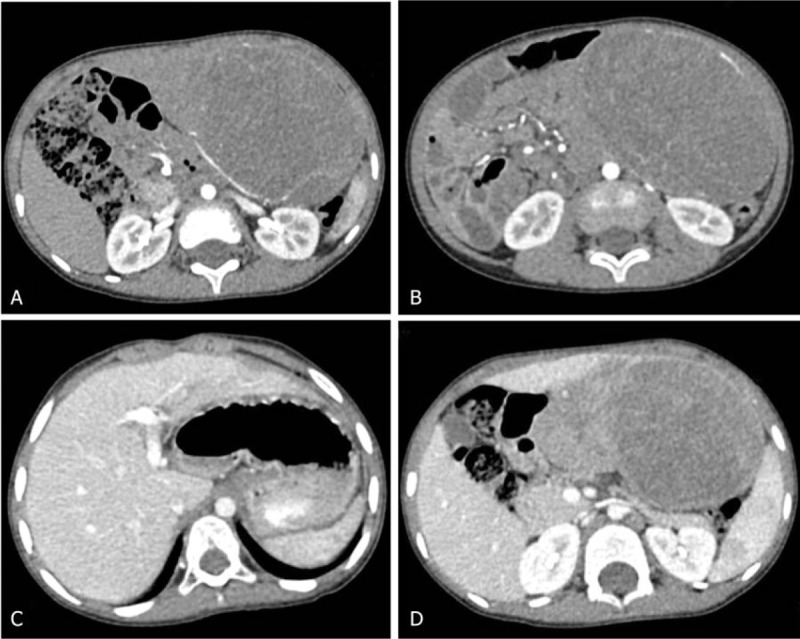
Preoperative abdominal CT scanning. (A) Arterial phase shows the edge of MHL is enhanced. (B) Arterial phase shows uneven nodular enhancement. (C) Portal vein phase shows left branch of the portal was pressed and distant part is not clear. (D) Portal vein phase shows uneven enhancement and the edge is clear.

A 4 and a half years old male child presented to our hospital for abdominal swelling. The child occasionally felt stomach ache that was relieved for no reason. The child showed no obvious tenderness and rebound. He was born as a premature baby in the 32nd week of his mother's pregnancy. His medical history included inguinal hernia on 2 sides, but he had only received an operation for the left inguinal hernia. No family or genetic history was found. Physical examination of this child showed that the abdomen was obviously bulging. The mass could be touched below umbilicus about 3 fingers. There were no other positive signs. The results of laboratory tests were almost normal, including alpha-fetoprotein (AFP) level. The Child–Pugh score was less than 5. Abdominal ultrasonography showed an uneven lesion in the liver and a diagnosis of HB was considered. Enhancement computed tomography (CT) scanning showed a large mass in the left upper abdomen and left lobe of the liver with an irregular edge. The size of the mass was 12.3 × 10.2 × 7.2 cm (Fig. 1). There was an uneven nodular shadow and the edge was obvious when the mass was enhanced. Calcification and an expanded bile duct were not observed. The left branch of the portal was pressed and the distant part was shown to be unclear. Abnormal was not observed in the right branch. According to the medical history, the diagnosis of HB was considered. We did not recommend a biopsy to the patient because it might cause bleeding and tumor diffusion. An operation was necessary.

**Figure 2:**
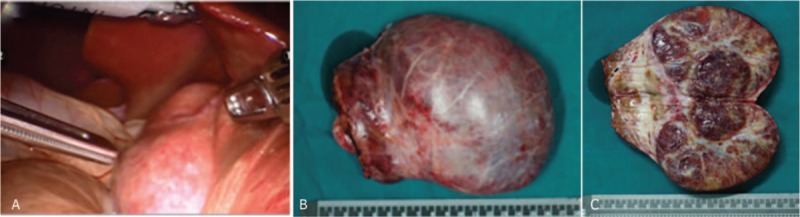
Tumor mass during operation. (A) An extrahepatic mass (12 × 10 cm) under DAVINCI robotic surgery. (B) The tumor in the left lateral hepatic lobe was removed. (C) The tumor edge is clear.

**Figure 3:**
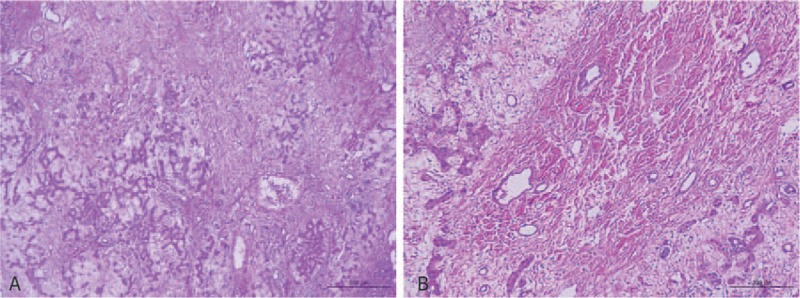
Pathological examination (100 × ). H&E staining shows edema mesenchymal, scattered liver cells, bile ducts, and cysts.

**Figure 4:**
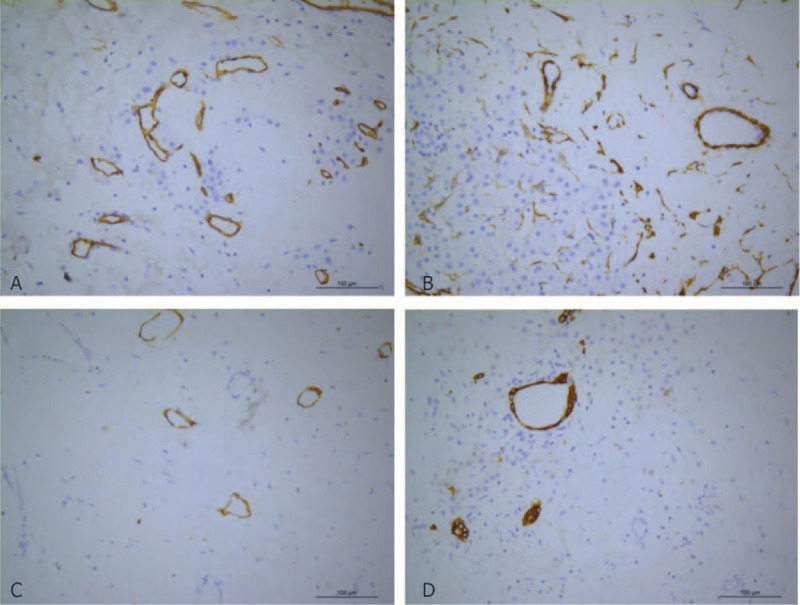
Pathological examination (200 × ). Immunohistochemistry staining shows mesenchymal CD34 (A), SMA (B), D2–40 (C), and epithelium CK19 (D) are positive.

The patient underwent DVSS surgery with sufficient preparation. Exploration showed an extrahepatic 12 × 10 cm mass in the left lobe of the liver (Fig. 2). After the operation, the sample was split. The edge of the tumor was clear but no envelope. Tremelloid mesenchyme was seen on the section (Fig. 2C). The pathological examination showed that the sample was grey red and 14 × 13 × 7 cm in size. The color of the section was grey red and grey white. MHL was diagnosed (Fig. 3). Immunohistochemistry results (Fig. 4) were the following: mesenchymal CD34 (+), D2–40 (+), S-100 (-), SMA (+), CD7 (-), epithelium CK19 (+), AFP (-), HCG (-). After 9 days of recovery, the patient returned home without complications. A follow-up examination, which included blood routine, liver renal function, coagulation routine, and ultrasound scan at 30 days, did not show any abnormality. No adverse or unanticipated event was presented.

## 3 Discussion

MHL was first reported in 1903 by Maresch.^[1]^ It also has other names such as cavernous lymph adenomatoid tumor, bile cell fibroadenoma, or benign mesenchymoma. Edmondson^[7]^ provided the first definite description of MHL. The incidence of MHL is 0.7 per million population per year.^[8]^ The precise pathogenesis of MHL is uncertain. Aberrant mesenchymal development in the portal tract, likely related to the bile ducts, is the most accepted theory.^[9,10]^ Some reports suggest that MHL has the potential to become embryonal sarcoma or angiosarcoma, though there have been no reported cases. Some researches have shown that the *C19MC* gene on chromosome band 19q13.4 has some bugs. A cluster of 46 microRNAs is encoded by the gene. Those microRNAs are normally only expressed from the paternal allele and in the placenta.^[11]^ In 2010, for a 2 years old female child with MHL, Sugito et al^[12]^ reported a chromosome test that showed t(11;19)(q13;q13.4).

MHL often occurs in children within 2 years old, with an average age of 15 months old. A slight male predominance (3:2 male:female ratio)^[13]^ is observed for MHL. Here, some literature related to MHL is discussed (Table 1).^[14–28]^ Approximately 80% (14/18) of patients were less than 2 years old. All patients had neither brothers nor sisters. Approximately 80% (14/18) came to hospital for mass or abdominal distention. Only 23% (4/17) of patients had mass in the left lobe of the liver. Only 2 patients had a high level of AFP. Only 28% (5/18) of patients got correct diagnosis before operation. Almost all patients had no special medical history. Typical clinical manifestations included painless and progressive bloating and abdominal mass. However, they usually do not become emaciated, in contrast to HB. A small number of patients had a high level of α-fetoprotein. The size of MHL varied from a few millimeters to more than 15 cm, or even 30 cm.^[13]^ Seventy-five percent of cases occurred in the right lobe of the liver. Ultrasound scans often showed uneven lesions, maybe for reasons of unique cystic mass. There was no rich blood flow sign because of a lack of vessels. Clear blood flow could not be seen at the end of portal or hepatic vein, perhaps because of repression. MHL showed no specific characteristic under unenhanced CT, whereas it showed multiple small cystic lesions in a solid mass in enhanced CT scan.^[14]^ The MR image of MHL is also diverse, that the characteristic of MR image depends on cysts, protein content in the fluid, and stromal composition of the mass.^[14]^ Because of liver fibrosis, solid areas of MHL may show hypointense both on T1 and T2-weighted images.^[14]^ No doctor used the nuclear medicine method to confirm this. The pathological features are the following: the surface of tumor is smooth and soft, and cysts were found on the section. Those cysts contain mucus. Hematoxylin and eosin (HE) staining shows various kinds of mesenchymal components such as expand lymphatic, vessel, irregular bile duct, myxoid matrix, and collagen. Some liver cells scatter but some cluster together. The tumor never becomes malignant and relapses after an operation. An immunohistochemical study showed spindle cells positive for vimentin and smooth muscle actin and negative for CD31, CD34, and S100 proteins, while the ducts stain positive for CK7 and negative for CK20.^[29,30]^

**Table 1:**
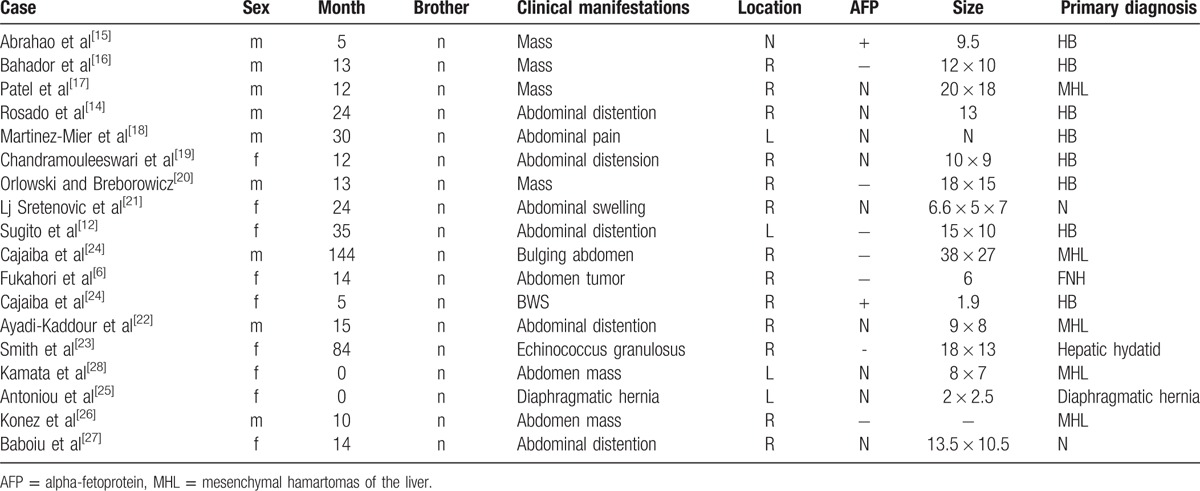
Literature review of reported MHL cases.

The characteristic of this case was that, this patient was more than 2 years old, in contrast to most reported cases. Moreover, this patient had an identical twin, while all cases of MHL in the literature did not involve any twins. The tumor was in the left lobe of the liver, while for most cases (13/17) they were in the right lobe. He had no high-level AFP, which suggested that HB was not likely. The enhancement CT showed some characteristic of MHL. An uneven nodular shadow and edge was obvious when the mass was enhanced. The feature of enhanced CT images matched with Swiss cheese appearance. It pointed to the way to MHL.

**Figure 5:**
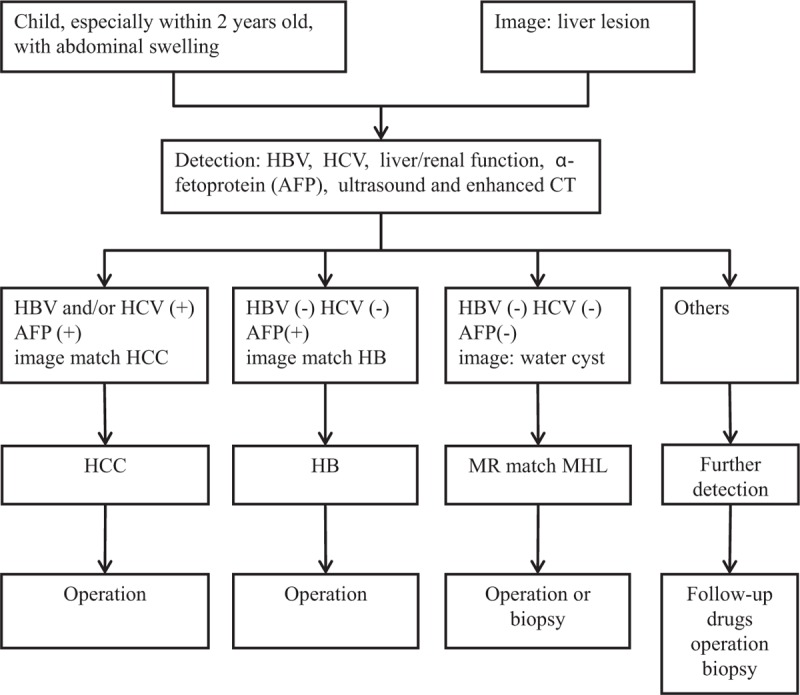
Diagram for diagnosis of tumor in the liver of children.

A limitation of the study is that we did not take MHL as a possible preoperative diagnosis because we lack experience. We propose the diagram for diagnosis of tumor in the liver of children (Fig. 5). If we find a mass in the liver of a child, we firstly consider it to be HB because this is the most common and harmful malignant liver tumor in children. The management of MHL remains controversial. MHL has the potential involute spontaneously, especially those with a component of prominent. Percutaneous aspiration may be helpful for large cysts that threaten life and give a chance to take an operation. Till now, complete resection is still the recommended way to treat with MHL. The method includes liver lobectomy and trisegmentectomy. The method should be determined by the location of the tumor and the technical skills of doctors in liver resection. For very large MHL, a large-scale liver resection may cause liver failure, and liver transplantation may be a good alternative. It is the same to the unresectable tumors.^[28,31]^

HB is the most common malignant liver disease in children. It often occurs in children within 3 years old, and it composed 50% to 62% of malignant liver lesions in children. HB patient often becomes emaciated, anemic, and anorexic. Liver function is usually not affected. AFP is often positive and the hepatitis B virus surface antigen is negative (Fig. 5). AFP increases or decreases with tumor growth or subsides in about one-third of patients (Table 2). To treat HB, surgical intervention is usually the first choice. Chemotherapy after surgical treatment should be because HB cells are usually sensitive to chemotherapeutic treatment.

**Table 2:**
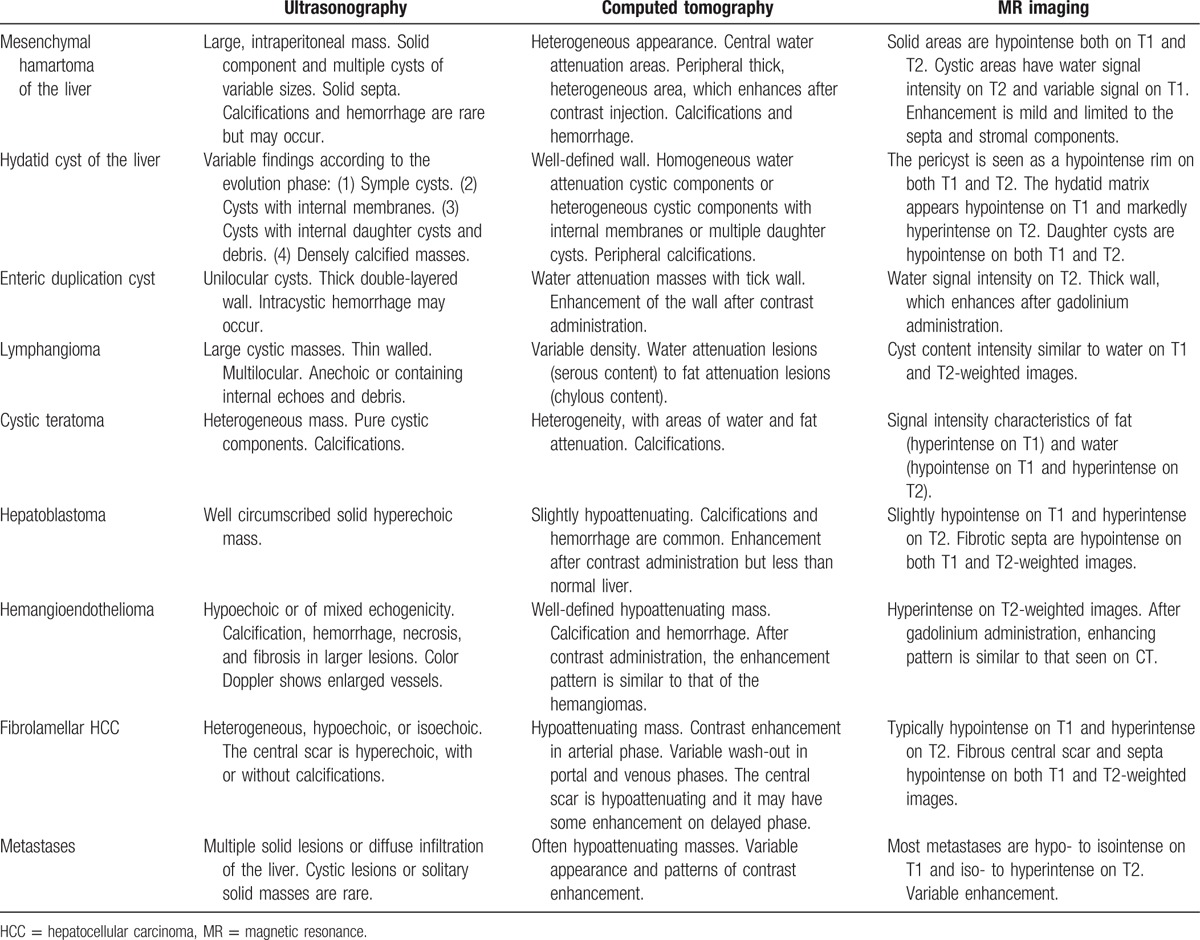
Imaging differential diagnosis between MHL and other types of liver mass.

HCC is the most common malignant liver disease for adults, but we also found a female infant (6 months old) in our hospital with HCC. About 90% of patients have a background of HBV (Fig. 5). The process is rapid. AFP is positive for about 70% of patients. The general condition is bad for most patients (Table 2).

In MHL, lack of typical clinical manifestations and characteristic image appearances make doctors difficult of diagnose it.

Some patients may show portal hypertension for the pressure of large MHL tumors. We summarized a distinguish table, which may help us to recognize it. The misdiagnosis of MHL usually shows a negative influence on patient's management. Most patients went to hospital for clinical manifestations induced by large tumors. Operations are necessary for such patients. If MHL patient was not able to or not willing to undergo liver resection, those clinical manifestations will be still or even worse. The tumor never becomes malignant. It does not relapse when it is removed totally.

## 4 Conclusion

This case suggests that surgeons should consider MHL as a possible diagnosis, although it is rare in more than 2 years old children, especially for those whose AFP is negative. MHL is a benign tumor that is hard to diagnose due to nonspecific clinical manifestations or examinations. The management of MHL remains controversial. Complete resection is recommended to treat MHL for good long-term outcomes. It is also suggested that parents take their children to hospital for examinations at least once a year.
